# A Case of Congenital Malignant Spinal Cord Glioma as a Cause of Congenital Ascites in a Neonate

**DOI:** 10.1155/2016/5208753

**Published:** 2016-08-14

**Authors:** Bianca Karber, Lenore Omesi, Sunny Chang, Andrew Handel, Monica Hegedus, Echezona Maduekwe

**Affiliations:** Stony Brook Children's Hospital, Stony Brook, NY 11794, USA

## Abstract

Congenital ascites is rare, but when it occurs, urinary ascites secondary to posterior urethral valve obstruction is the most common, and tumors are the least. Among the tumors in the pediatric population, the central nervous system tumors are common, but spinal cord tumors are rare. We describe a very rare case of congenital malignant spinal cord glioma presenting as isolated congenital ascites secondary to neurogenic bladder. A female infant was diagnosed sonographically with isolated congenital ascites at 40 weeks' gestational age, with uneventful development prior to 40 weeks' gestational age. Magnetic resonance imaging of the spine done within the first week of life identified a lobulated spinal mass with heterogeneous enhancement within the conus medullaris. Spinal fluid analysis showed evidence of small round blue cells and the pathology from the excision biopsy of the mass confirmed a WHO grade III or IV malignant glioma. The postoperative course was uneventful with resolution of the ascites and spontaneous micturition. The patient was discharged home without an indwelling urinary catheter. We report the first documented case of a newborn infant with isolated congenital ascites from neurogenic bladder secondary to a spinal cord glioma.

## 1. Introduction

Tumors of the central nervous system are common in the pediatric population, constituting the second most prevalent tumor type in children. Spinal cord tumors account for 1–10% of all pediatric central nervous system tumors [1–3]. However, only 2% of childhood tumors have been reported to appear in the neonatal period [4].

Neonatal tumors are more often benign and rarely present with urinary ascites. We report a first documented case of a congenital malignant spinal cord glioma.

## 2. Case Report

**Figure 1:**
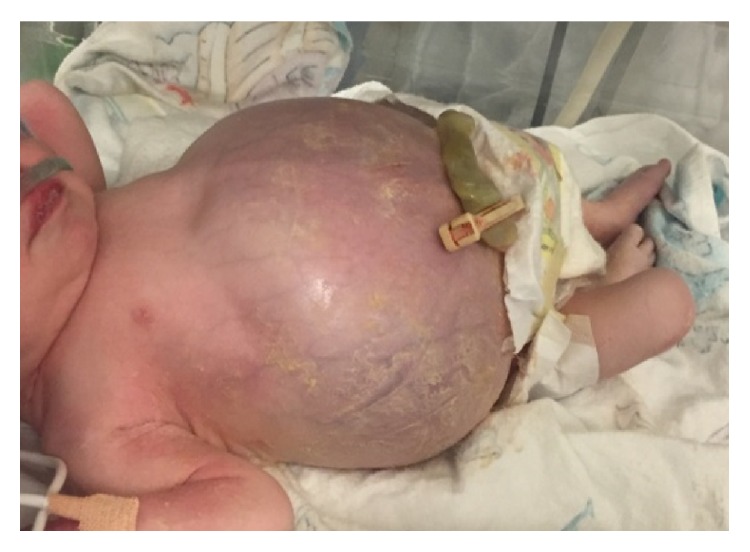
Neonate soon after birth: physical exam suggestive of massive ascites.

A full-term 3.8 kg female of 40 weeks' gestation was born via C-section for isolated fetal ascites seen on sonogram. The prenatal course history was significant for conception through in vitro fertilization using a donor egg, anhydramnios, and isolated congenital ascites seen on sonogram at 40 weeks' gestational age. This repeat sonogram was done due to failed induction three days prior to delivery, after having had a normal sonogram four weeks earlier. At delivery, the baby was tachypneic and had a tense shiny distended abdomen (see Figure 1). She required minimal resuscitation and was given nasal CPAP prior to transfer to the neonatal intensive care unit (NICU). Apgar scores were 7 and 8 at 1 and 5 minutes, respectively.

**Figure 2:**
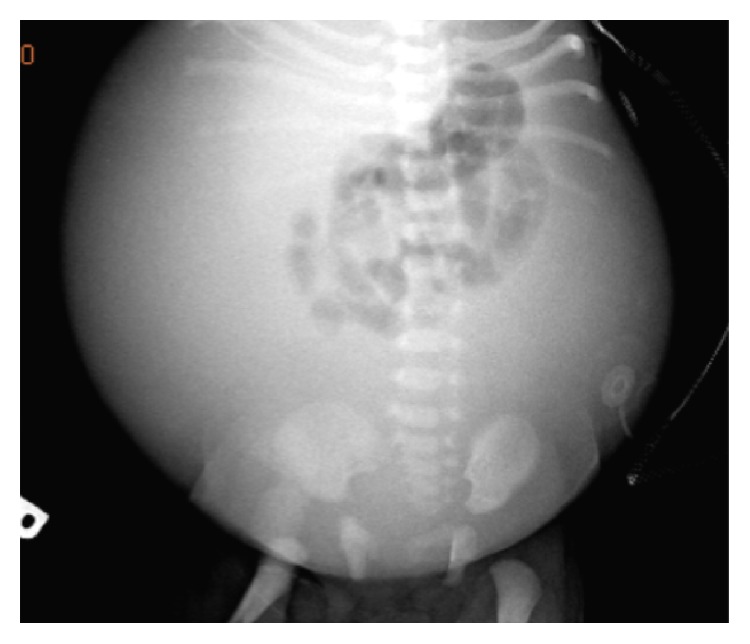
Abdominal X-ray, day of life 1: distended abdomen with centralization of bowel loops showing massive ascites.

**Figure 3:**
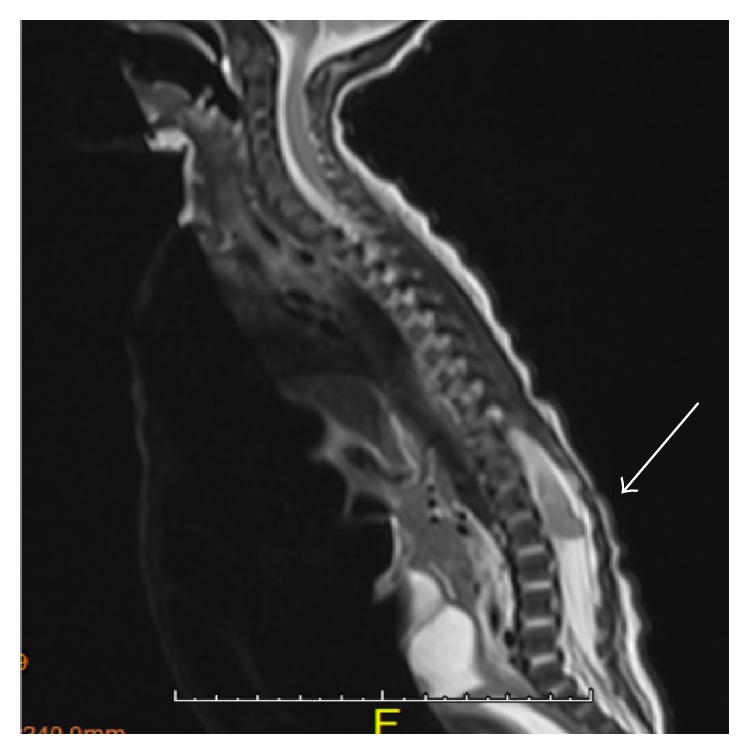
MRI spine, with and without contrast: expansile, somewhat lobulated mass in conus medullaris (see arrow).

**Figure 4:**
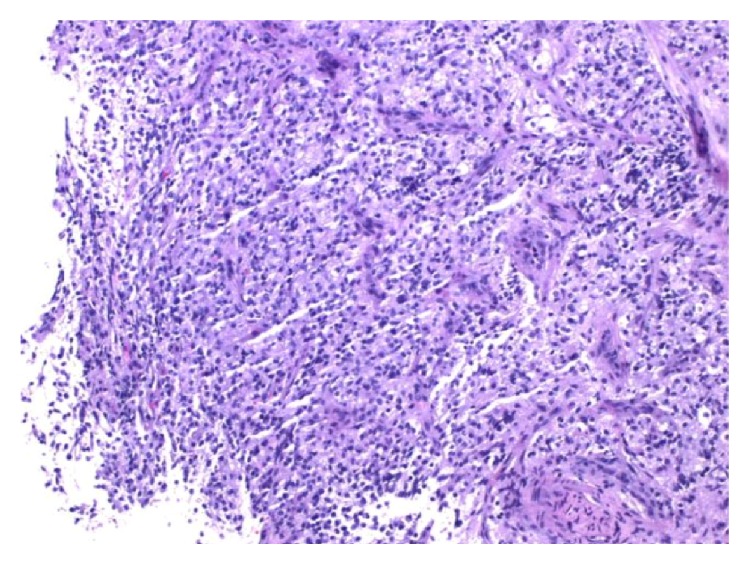
Surgical pathology: malignant neoplasm most consistent with malignant glioma.

Abdominal X-ray and ultrasonography in the NICU confirmed massive ascites with centrally floating bowel (see Figure 2) with ultrasound reporting no hydronephrosis and a decompressed bladder. The kidneys were normal. Chylous ascites was ruled out on analysis of the peritoneal fluid which showed no lymphocytes. However, the creatinine, in the peritoneal fluid, was higher than the serum creatinine, suggestive of urinary ascites [4]. The patient's admission labs and peritoneal fluid analysis are available in the Appendix. Adequate urine output was only noted on the aspiration of an indwelling urinary catheter with a syringe, which was suggestive of neurogenic bladder. Cystography and cystoscopy did not show any evidence of vesicoureteric reflux or urinary leakage from the urinary system, and hydronephrosis was evident on a repeat renal sonogram. Further diagnostic investigation with an abdominal MRI demonstrated a spinal mass. A subsequent focused MRI evaluation of the brain and spine confirmed a lobulated mass with heterogeneous enhancement within the conus medullaris measuring 0.9 × 1.4 × 0.8 cm (see Figure 3). Spinal fluid analysis after MRI showed multiple small round blue cells. The mass was identified as WHO grade III/IV malignant glioma on excision biopsy (see Figure 4). The hydronephrosis resolved with continuous draining of urine through the indwelling urinary catheter which was eventually discontinued during her NICU course as she started voiding spontaneously after the subtotal excision biopsy. She was discharged home and is currently undergoing experimental chemotherapy for the glioma.

## 3. Discussion

Isolated neonatal ascites defined as ascites without hydrops is rare. When it occurs, the most common cause is urinary ascites with urinary obstruction accounting for approximately 60 to 70% of cases, isolated fetal chylous ascites accounts for 4 to 20% of the cases, and hepatobiliary and intestinal causes account for 15 to 30% [5].

The most common cause of the urinary obstruction is posterior urethral valves and less commonly urethral atresia. All documented cases of urinary ascites in the literature are from prenatally diagnosed bladder rupture and all were in male infants except one, a female diagnosed in utero with spontaneous bladder rupture of unknown etiology [6]. There were two documented cases of urinary ascites due to spinal cord pathology: one was a case of bladder rupture and dysfunction secondary to neurogenic bladder from a sacrococcygeal teratoma [7] and the other as a result of complication from surgical repair of myelomeningocele. Mann et al. also reported a case of urinary ascites from bladder rupture secondary to a congenital neuroblastoma with complete block of the spinal canal in the lumbar region [8]. Our patient had urinary ascites with no evidence of bladder rupture. The origin of the urine might be from breaks in the bladder or at the level of calyceal fornices not seen on ultrasound or it may have been from transudation of urine from an intact urinary tract [7]. The most acceptable theory for urinary ascites caused by a neurogenic bladder is that extrinsic compression by a mass can disrupt nerve connections to the bladder resulting in bladder atonia and eventual rupture.

When congenital urinary ascites occurs in neonates, it can be complicated by electrolyte disturbances including hyponatremia, hyperkalemia, and elevated creatinine as a result of “autodialysis” [8] when urine is in contact with peritoneal membrane. These complications were not noted in our patient, perhaps due to acute recent onset of anhydramnios with little time for serum changes to occur.

Spinal cord tumors are rare in the neonatal period, with conus medullaris tumors being even rarer among this category. The most common neonatal tumors are teratomas which are most likely extragonadal and mainly present in the sacrococcygeal or mediastinal area. Neurologic features of spinal cord tumors in the lumbosacral-coccygeal area include lower extremity weakness and disturbance of sphincter function [9]. All previously reported 24 cases of congenital malignant glioma involved the brain (19 supratentorial cases, 1 infratentorial case, and 2 cases with locations not given) [10]. Among intraspinal neoplasms in the pediatric age group, the most frequently noted were astrocytoma (47%) and the ependymal neoplasm (24%) [11]. The median age at diagnosis was 10 years with a male-to-female ratio of 1 : 1.

Our case, therefore, provides documentation of the youngest infant with malignant spinal cord glioma. Although it is a rare condition, a diagnosis of spinal cord tumor should be considered in neonates with congenital ascites and neurogenic bladder. Despite advances in imaging technology to diagnose spinal cord tumors in neonates, the treatment modalities have not had the same significant gains. Current treatment is still experimental chemotherapy, as is the case with our index patient.

## Figures and Tables

**(a):** 

Basic metabolic panel	Values	Normal range
Sodium (mmol/L)	138	135−148
Potassium (mmol/L)	5.2	3.5−5.1
Chloride (mmol/L)	103	98−108
Bicarbonate (mmol/L)	23	21−31
Glucose (mg/dL)	83	50−80
BUN (mg/dL)	11	5−20
Creatinine (mg/dL)	0.23	0.5−1.2
Calcium (mg/dL)	9.8	8.6−10.2
Phosphorus (mg/dL)	6.4	4.5−6.7

**(b):** 

Complete blood cell count/differentials	Values	Normal range
WBC count (×10^3^/*μ*L)	17.77	7.0−17
RBC count (×10^6^/*μ*L)	3.03	3.46−6.26
Hemoglobin (g/dL)	9.7	14.6−20.1
Hematocrit (%)	30.1	50−69
MCV (fL)	99.3	96−120
MCH (pg)	32.0	27−31
MCHC (g/dL)	32.2	33−37
RDW (%)	16.3	11.2−14.8
PLT count (×10^3^/*μ*L)	243	150−350
MPV (fL)	10.4	8.0−12
Neutrophil (%)	51	
Band (%)	2	0−15
Eos (%)	3	0−3
Lymphocyte (%)	30	

**(c):** 

Peritoneal fluid analysis	Values
Color	Yellow
WBC count (per *μ*L)	11
RBC count (per *μ*L)	18
Neutrophil (%)	16
Mononuclear (%)	68
Eosinophils (%)	16
Albumin (g/dL)	1.6
BUN (mg/dL)	13
Cholesterol (mg/dL)	35
Creatinine (mg/dL)	0.4
LDH (IU/L)	141
pH	8.0

## Appendix

See Table 1.
